# A participatory rapid ethnographic assessment protocol for studying human-animal-material interactions in veterinary clinical practice

**DOI:** 10.3389/fvets.2026.1891996

**Published:** 2026-08-12

**Authors:** Brandon W. Kliewer, Ron Orchard

**Affiliations:** 1 Leadership Studies, University of Tennessee, Knoxville, TN, United States; 2 College of Veterinary Medicine, Kansas State University, Manhattan, KS, United States

**Keywords:** clinical education, community engagement, One Welfare, participatory rapid ethnographic assessment, veterinary coaching

## Abstract

Veterinary clinical practice involves complex interactions among clinicians, learners, clients, animals, material objects, and built environments. These interactions shape how care is delivered, how students learn, and how practitioners respond to changing circumstances in real time. However, existing qualitative and ethnographic methods often lack a replicable protocol for capturing how human, animal, and material contributions jointly shape clinical practice as it unfolds. This methodology article presents a participatory Rapid Ethnographic Assessment protocol designed for veterinary educators, practitioners, and researchers who seek to study, teach, and improve context-sensitive clinical practice in interspecies settings. The protocol details the epistemological and philosophical implications of a two-camera audiovisual data collection strategy: one camera records naturally occurring clinical interactions for later research analysis, while a second assessment camera captures interactional sequences that can be rapidly replayed to participants for reflection and coaching. The method proceeds through four stages: identifying candidate sequences of practice, conducting participatory interactional sense- and meaning-making, organizing paired assessment and research recordings for close analysis, and interpreting selected sequences using relevant clinical, educational, organizational, or social science theory. The protocol is illustrated through an example from mobile veterinary care in which a dog treat, clinician, client, patient, and technician collectively contribute to redirecting an interaction and enabling care to proceed. Anticipated outputs include participant-validated interactional data, teachable examples of clinical and leadership practice, context-specific quality improvement insights, and evidence that can inform veterinary education and One Welfare-oriented outreach. The article details required materials, step-by-step procedures, timing, pause points, anticipated results, limitations, pitfalls, and troubleshooting strategies. By making human-animal-material interactions visible and analyzable, this methodological protocol offers a practical method for studying and improving clinical practice in veterinary humanities, social science, and community-engaged veterinary research.

## Introduction

Veterinary clinical practice is produced through complex interactions among clinicians, clients, students, technicians, animal patients, clinical tools, built environments, institutional routines, and time-sensitive decisions. Clinical care rarely unfolds as a linear application of technical knowledge. Instead, veterinarians and veterinary teams must continuously interpret patient behavior, negotiate client concerns, coordinate with colleagues, manage equipment and space, and adapt care plans to the circumstances of the encounter. These dynamics are particularly visible in teaching hospitals, general practice, emergency and critical care, shelter medicine, mobile clinics, farm animal practice, and community-based outreach, where clinical, educational, ethical, and relational demands often converge in real time.

The social and communicative dimensions of veterinary medicine are now well recognized. Communication is central to history-taking, consent, treatment planning, adherence, client trust, and shared decision-making in veterinary practice (1, 2). Veterinary education scholars have also emphasized that clinical competence is learned not only through formal instruction but through situated, workplace-based participation in real clinical environments where students learn through observation, supervised practice, feedback, reflection, and increasing responsibility (3, 4). Competency-based veterinary education has further clarified the importance of observable professional activities, entrustment, communication, clinical reasoning, collaboration, professionalism, and professional identity formation as core dimensions of clinical development (5, 6, 31). Qualitative approaches have therefore become increasingly important for understanding how students, clinicians, and clients make sense of veterinary care in practice (7). Qualitative data collection, situated in real-time allows for context specific knowledge and understanding of practice (8). However, many existing approaches rely on interviews, surveys, retrospective reflection, assessment documentation, or isolated observation. These methods provide valuable evidence about perceptions, experiences, and outcomes in veterinary education and practice (7), but they are less able to capture how clinical learning, coaching, communication, animal behavior, and material conditions are organized moment by moment during authentic veterinary encounters. Video-reflexive and interactional approaches are better suited to making such situated clinical work visible for participant interpretation and analysis (9–11). Post-positivist qualitative methods collect forms of empirical data helpful for unpacking individual and group-level recollections and perspectives of socio-political phenomenon. Clinical veterinary medicine practice could benefit more from qualitative methods that rely on observable interactions.

Collecting naturally occurring qualitative data responds to a broader methodological challenge associated with studying and developing veterinary practice see Table 1. For one, veterinary medical practice is not only interpersonal but also includes inter-species and socio-material interactions. Animal patients do not simply receive care passively. Their movements, vocalizations, posture, affective states, pain responses, resistance, cooperation, and learned behaviors can alter the flow of clinical work. Similarly, material objects such as leashes, muzzles, treats, examination tables, scales, stethoscopes, consent forms, cages, electronic medical records, and mobile clinical infrastructure help shape what forms of care become possible in a given moment. Human-animal studies and multispecies scholarship have shown that animal-human relations can influence trust, knowledge, identity, and social organization (12–14, 30). In veterinary settings, however, researchers and educators still need practical and replicable methods for documenting how these human, animal, and material interactions shape clinical practice as it unfolds.

**Table 1:** Anticipated outputs and uses.

Pitfall or artifact	Likely effect	Troubleshooting measure
Poor audio quality	Coaching or clinical dialogue cannot be analyzed reliably	Use external microphones; test audio before recording; position camera closer to speakers without disrupting care
Poor camera angle	Animal behavior, object use, or participant action is missed	Pilot camera placement; use wide-angle view; adjust placement by room type
Recording disrupts clinical care	Participants become distracted or patient care is affected	Use small unobtrusive equipment; stop recording when care requires; prioritize patient welfare
Participants perform for the camera	Data may reflect altered behavior	Conduct familiarization sessions; record multiple encounters; note possible reactivity in fieldnotes
Playback becomes evaluative or punitive	Participants may become defensive or reluctant	Frame playback as learning and reflection; use open-ended prompts; avoid blame-oriented language
Coach dominates interpretation	Participant validation is weakened	Ask participants to describe what they notice before offering feedback
Client discomfort	Consent may be compromised or participation reduced	Use clear consent procedures; allow opt-out; stop recording or playback on request
Animal distress	Welfare and safety concerns	Stop recording; follow clinical handling protocols; resume only if appropriate
Over-attribution of agency to animal or object	Analysis becomes speculative	Describe observable behavior and material effects; avoid claims about intention unless supported
Data overload	Too much video becomes unmanageable	Select short candidate sequences; use case logs; define analytic priorities before collection
Privacy breach risk	Ethical and institutional concerns	Use encrypted storage; restrict access; remove identifiers from transcripts; follow data retention policy
Coaching footage not linked to clinical footage	Paired analysis becomes difficult	Use standardized file naming, case logs, and time stamps immediately after recording

This table could list each output—video sequence, coaching transcript, analytic memo, coaching code, quality improvement insight—and identify its use for research, education, or practice improvement.

One Welfare and One Health frameworks further strengthen the need for methods that can follow the interdependence of human, animal, and environmental wellbeing in practice. One Welfare extends attention beyond disease control or individual animal care to the reciprocal relationships among animal welfare, human wellbeing, social conditions, and environmental contexts (15–17). For veterinary humanities and social science research, this creates a methodological need: researchers must be able to study veterinary clinical encounters as situated practices in which clinical decisions, educational moments, client relationships, animal behavior, and material arrangements are mutually influential. A method suited to this task must be close enough to everyday clinical work to capture interactional detail, but structured enough to generate analyzable and replicable data.

Rapid ethnographic approaches offer one response to this challenge. Rapid ethnographic assessment is designed to generate context-sensitive qualitative insight within practical time frames, particularly in applied settings where researchers, practitioners, and communities need timely knowledge to support decision-making or improvement (18). Rapid ethnographic approaches are particularly well suited to be organized around onto-epistemological assumptions attempting to uncover knowledge and understanding of practice that exists between process and socio-material interactions (19). The underlying philosophical assumptions of Rapid Ethnographic assumptions allow for meaningful analysis of fine-grain interactions that enact emergence of process and practice (8). Related approaches in health services research, including rapid assessment procedure-informed clinical ethnography, have been used to study implementation, organizational context, and clinical work in ways that remain sensitive to local practice conditions (20). These approaches are useful for veterinary settings because they allow researchers to examine practice in real-world environments without requiring long-term immersion that may be impractical in busy clinical contexts.

Video-based and video-reflexive methods are also relevant. In human healthcare, video reflexive ethnography has been used to make everyday clinical work visible to practitioners, support collective reflection, and identify opportunities for practice improvement (9, 10). Video-reflexive methods are especially useful because they do not treat video merely as a recording device; rather, video becomes a prompt for practitioner sense-making, reflexive learning, and local change. Studies of medical trainees and clinical teams have shown that video-reflexive approaches can help participants notice tacit features of practice, including coordination, safety, communication, and workplace learning (21, 22). Veterinary education and clinical practice can benefit from similar approaches, but veterinary contexts add distinctive methodological demands because the animal patient is both a recipient of care and an active participant in the interaction.

The methodological gap addressed by this article is therefore not the absence of qualitative, ethnographic, video-based, or workplace-learning research. Rather, the gap is the lack of a structured and replicable protocol for linking three forms of evidence in veterinary clinical settings: naturally occurring clinical interaction, rapid participant interpretation of that interaction, and the coaching or reflexive dialogue through which clinical meaning is negotiated. This linkage is important because key constructs in veterinary education, clinical judgment, communication, animal handling, feedback, reflective learning, teamwork, and professional identity formation, are not only individual attributes or retrospective perceptions. They are also enacted interactionally through encounters among students, clinicians, clients, animal patients, tools, spaces, and institutional routines. A protocol capable of capturing these interactions and returning them to participants for sense- and meaning-making can therefore strengthen the empirical study of clinical learning while also supporting context-sensitive coaching and practice improvement.

This article presents a participatory Rapid Ethonographic Assessment protocol for studying human-animal-material interactions in veterinary clinical practice. The protocol combines rapid ethnographic assessment with audiovisual recording, structured participant reflection, and interactional sense- and meaning-making. Its central innovation is a two-camera design. The first camera records naturally occurring clinical interactions and provides the footage used for rapid playback during coaching and reflexive assessment. Shortly after a selected interaction occurs, this footage is reviewed with relevant participants, such as clinicians, students, technicians, clients, or educators, to support reflection on what happened, how the interaction unfolded, and how human, animal, and material elements shaped the clinical encounter. The second camera records this playback, coaching, and reflexive sense-making process as research data. In doing so, it also captures how veterinary coaching is enacted in practice including the questions, prompts, interpretive moves, feedback strategies, and teaching interventions used to help participants make sense of clinical action. This dual-recording structure allows the protocol to capture both the clinical interaction itself and the situated coaching practices through which participants interpret, operationalize, and refine veterinary clinical practice soon after it occurs. As a qualitative methodology, the dual-recording structure collects naturally occurring data useful in understanding interactive processes and practices in clinical settings. The goal of highlighting the ontological and epistemological assumptions of collecting naturally occurring data, is to showcase the range of assessment and research application.

Research and clinicians must situate the methodological protocol within four broad stages. First, researchers identify candidate sequences of clinical practice in which human, animal, or material interactions appear to shape the direction of care, communication, learning, or coordination. This could include an range of unit of analyzes to be chosen by practitioners and researchers. For our purpose of establishing this methodology in human and social sciences veterinary research, we encourage candidate sequences that focus on dynamics of human-animal-material agency that informs the clinical experience. This could include a range of units of analysis to be chosen by practitioners and researchers. Establishing this methodological protocol encourages veterinary humanities and social sciences to explore candidate sequences that are culturally and contextually situated and focus on dynamics associated with human-animal-material interactions in a clinical veterinary medicine setting. Second, selected sequences are used for participatory reflection and interactional validation, allowing participants to explain what they noticed, intended, experienced, or adjusted during the encounter. Third, the research and assessment recordings are paired and organized for close analysis. Fourth, selected sequences are interpreted using analytic frameworks appropriate to the study purpose, such as veterinary communication, clinical education, trauma-informed practice, workplace learning, One Welfare, implementation science, interaction analysis, or organizational change theory. This design is intended to support both research and practice development by connecting observation, participant interpretation, and applied learning.

The broad methodological protocol can be used in a range of veterinary clinical settings that is consistent with the processual ontology and epistemological assumptions of leadership and clinical practice. In veterinary education, it can help faculty and learners examine how students develop clinical judgment, communication, animal handling, professional identity, and team coordination. In clinical practice, it can help teams identify recurring interactional patterns that influence efficiency, patient welfare, client communication, or safety. In shelter, outreach, or community-based medicine, it can help researchers and practitioners study how care is shaped by social vulnerability, material constraints, public health concerns, and human-animal relationships. In quality improvement work, it can help teams generate locally grounded evidence about how specific clinical practices are enacted and how they might be refined.

The purpose of this Methods article is therefore to provide a replicable protocol for researchers, practitioners, and educators, who seek to study veterinary clinical practice as it happens. The article describes the materials and equipment required, step-by-step procedures, timing, pause points, validation strategies, anticipated data outputs, expected results, limitations, potential pitfalls, and troubleshooting measures. By making human-animal-material interactions visible and analyzable, the protocol offers a practical method for studying and improving context-sensitive veterinary clinical practice.

## Materials, equipment, and ethical considerations

The required materials consist of audiovisual recording equipment, playback equipment, consent and documentation materials, secure data-management tools, and clinical or educational materials already present in the veterinary setting. Equipment should be adapted to the clinical environment, species, and institutional privacy requirements. Of course, appropriate institutional review board and ethics policies must be followed. However, participatory rapid ethnographic assessment methods that blur the lines between subject and object of study require additional social, ethical, and political considerations (23). Leadership educators should closely attend to the circumstances of power, identity, and systems that show up in various clinical processes and practices being considered. Because the protocol is intended for real-world veterinary clinical practice, equipment should be portable, minimally disruptive, easy to clean or disinfect, and safe for use around animal patients.

### Audiovisual recording equipment

The protocol requires two video-recording devices. Camera 1 records the naturally occurring veterinary clinical interaction and produces the footage used for rapid playback, coaching, and reflexive assessment. Again, in our case, we use this methodology in the context of a mobile shelter medicine program. However, the methodological protocol can be adjusted to include a range of veterinary medicine settings. Camera 2 records the playback, coaching, and participant sense-making session. The original manuscript frames this two-camera approach as central to linking clinical interaction data with reflection, coaching, and analysis of practice. Recordings from Camera 2 support increased clinical and leadership learning that is connected directly to context. This empirical data can support a range of learning and development based on the needs of the practitioner or research context.

Suitable recording devices include digital video cameras, tablet cameras, smartphones with high-resolution video capability, or institutionally approved clinical recording systems. Devices should be able to record both video and audio clearly for the expected duration of the encounter. When possible, Camera 1 should use a wide-angle lens or be positioned to capture the clinician, learner, client, animal patient, relevant objects, and clinical space. Camera 2 should be positioned to capture the playback session, including the coach/facilitator, participants, and verbal discussion.

Recommended recording equipment includes:

- Two video-recording devices with sufficient battery life and storage capacity.
- Tripods, tabletop mounts, mobile stands, or other stable supports.
- External microphones, lapel microphones, or boundary microphones when room audio is poor.
- Headphones or small speakers for playback review, if needed.
- Charging cables and backup battery packs.
- Spare memory cards or approved encrypted storage media.
- Protective cases or mounts suitable for clinical environments.
- Disinfectable covers or wipeable equipment surfaces, where appropriate.

In high-noise environments, such as emergency treatment areas, barns, shelters, or mobile clinics, external microphones are strongly recommended. In confined examination rooms, a single fixed wide-angle camera may be sufficient for Camera 1, while Camera 2 may be placed near the playback device to record the coaching conversation.

### Playback equipment

The protocol requires a device that allows Camera 1 footage to be reviewed shortly after the selected interaction. The playback device should be portable, easy for participants to view, and approved for use with clinical or research data.

Playback equipment may include:

- Tablet, laptop, or secure clinical workstation.
- Video playback software that allows pausing, replaying, and reviewing short clips.
- Privacy screen, where needed.
- Headphones or low-volume speakers.
- Adapter cables, card readers, or secure transfer tools.
- A quiet review location, such as a teaching room, office, consultation room, or designated clinic space.

The playback setup should allow the coach/facilitator to pause the video at key moments and invite participants to describe what they notice before moving into interpretation or feedback.

### Documentation materials

Documentation materials are required to support consent, fieldnotes, sequence identification, case tracking, and later analysis. These materials should be prepared before data collection begins.

Recommended documentation materials include:

- Participant information sheets.
- Consent forms for clinicians, students, staff, educators, and clients.
- Site permission forms or clinical governance approval, where required.
- Animal patient recording and welfare documentation, consistent with institutional policy.
- Fieldnote template.
- Candidate sequence identification sheet.
- Case log linking Camera 1 and Camera 2 recordings.
- Playback/coaching prompt sheet.
- Adverse event or interruption log.
- Data access and withdrawal request form.
- Confidentiality agreement for research or education team members.

The case log should include the date, site, encounter type, participant roles, patient species, Camera 1 file name, Camera 2 file name, selected sequence time stamps, playback timing, consent restrictions, and preliminary analytic notes.

### Coaching and reflexive assessment materials

Because this protocol is also designed to codify and operationalize veterinary coaching, coaching materials should be standardized enough to support comparison across cases while remaining flexible enough for real clinical teaching. There are a range of definitions of leadership coaching across the leadership studies literature. However, generally leadership coaching is understood to be an intervention designed to develop leader/leadership capacity, decision-making, and the overall social capital of a group to accomplish leadership work in organizations (24–27).

Recommended coaching materials include:

- A short set of open-ended coaching prompts.
- A checklist for human-animal-material interaction features.
- A coaching observation template.
- A form for recording identified teaching points.
- A form for documenting possible quality improvement implications.
- Optional rubric for classifying coaching moves.

Examples of coaching prompts include:

- “What do you notice happening in this moment?”
- “What were you trying to accomplish clinically?”
- “How did the animal patient's behavior shape your next action?”
- “How did the client influence what happened?”
- “What role did the tool, object, room, leash, scale, treat, or other material feature play?”
- “What would you repeat in a similar case?”
- “What might you adjust next time?”

These prompts should be treated as flexible guides rather than a script. The purpose is to support participant reflection, interactional validation, and identification of teachable clinical practice.

### Data storage and analysis materials

The protocol produces identifiable audiovisual data and therefore requires secure data-management systems. Video-reflexive and video-based clinical methods raise important issues of consent, privacy, storage, and participant control over recordings; these concerns should be addressed before data collection begins.

Required data-management materials include:

- Encrypted institutional storage or secure research server.
- Password-protected devices.
- Secure file-transfer method.
- File-naming convention.
- Access-control log.
- Backup procedure.
- Data retention and deletion schedule.
- Transcription software or service approved for sensitive data.
- Qualitative analysis software, if used, such as NVivo, ATLAS.ti, MAXQDA, Dedoose, or an institutionally approved equivalent.
- Spreadsheet or database for case tracking.
- Transcription conventions for verbal, embodied, animal, and material actions.

Because video de-identification is difficult, raw video should be accessible only to authorized team members. Transcripts and analytic summaries should use role labels rather than names whenever possible.

### Clinical and environmental materials

The protocol does not require specialized veterinary clinical equipment beyond what is already used in the encounter under study. However, because the method examines how tools and objects shape clinical practice, researchers should document relevant materials present in the setting.

Depending on the case, these may include:

- Leashes, harnesses, carriers, cages, towels, blankets, muzzles, or restraint aids.
- Examination tables, scales, treatment surfaces, or mobile clinic equipment.
- Stethoscopes, thermometers, syringes, medications, bandaging materials, or diagnostic tools.
- Treats, food rewards, toys, or other animal-handling aids.
- Electronic medical records, consent forms, discharge instructions, or treatment plans.
- Room layout, doorways, seating, counters, kennels, and other spatial features.

If treats, toys, towels, or other handling aids are used, they should be consistent with clinical policy, patient safety, dietary restrictions, allergy concerns, and client consent. These items are not “reagents” in the laboratory sense, but they may become analytically important because they can shape the flow of clinical action.

### Cleaning and infection-control materials

Recording equipment should not compromise biosecurity or infection-control procedures. Equipment should be cleaned according to institutional policy and should not be placed on contaminated surfaces unless protected.

Recommended cleaning and safety materials include:

- Disinfectant wipes approved for the clinical setting.
- Disposable equipment covers, where appropriate.
- Hand sanitizer.
- Sealable bags or cases for transporting equipment.
- Labels distinguishing clean and used equipment.
- Species- or setting-specific biosecurity supplies, where required.

Researchers should confirm that disinfectants are compatible with recording devices and mounts. If equipment cannot be safely disinfected, it should be kept outside direct patient-contact zones.

### Optional materials

Optional materials may improve data quality or educational use:

- Wide-angle lens attachment.
- Secondary audio recorder.
- Time-synchronization tool or visible time marker.
- Portable lighting, if it does not disturb the patient.
- Whiteboard or digital note-taking tool for coaching sessions.
- Secure cloud-based qualitative analysis platform.
- Faculty development guide for coach training.
- Template for converting findings into quality improvement actions.

## Methods

### Objectives of the protocol

This protocol is designed to support the systematic study, teaching, and improvement of veterinary clinical practice by capturing how clinicians, learners, clients, animal patients, material objects, and clinical environments shape care as it unfolds. The method has four primary objectives: first, to record naturally occurring veterinary clinical interactions; second, to use those recordings as a prompt for rapid coaching, reflection, and interactional validation; third, to record the coaching and reflexive sense-making process so that veterinary coaching itself can be analyzed and operationalized; and fourth, to generate participant-validated qualitative data that can inform veterinary education, clinical communication, human-animal interaction research, One Welfare oriented practice, and quality improvement.

The protocol adapts Rapid Ethnographic Assessment for use in veterinary clinical settings and incorporates principles from video-reflexive ethnography, interaction analysis, workplace learning research, and veterinary qualitative inquiry (7, 9, 10, 18, 22). The method is intended for real-world clinical environments where extended ethnographic immersion may be impractical but where researchers, educators, or practitioners need close, context-sensitive evidence about clinical practice.

### Study design

The protocol uses a participatory, two-camera Rapid Ethnographic Assessment design. Camera 1 records naturally occurring veterinary clinical interactions. These recordings serve two purposes: they provide primary audiovisual data of the clinical encounter and they function as a rapid playback tool for coaching and reflexive assessment. Camera 2 records the playback, coaching, and participant reflection that follow selected clinical interactions. This second recording captures how supervisors, educators, clinicians, students, technicians, clients, or other participants interpret the clinical interaction, identify what mattered, and articulate how veterinary practice might be refined.

This design produces two linked data streams:

1. Clinical interaction data, consisting of Camera 1 recordings of the veterinary encounter.
2. Coaching and reflexive assessment data, consisting of Camera 2 recordings of participant review, coaching dialogue, interpretive discussion, and validation.

The paired data structure allows researchers to analyze both the original clinical interaction and the subsequent process through which participants make sense of that interaction. It also enables veterinary educators to codify coaching moves, including the questions, prompts, feedback strategies, interpretive cues, and teaching interventions used to support clinical learning.

### Recommended clinical contexts, focal cases, and units of analysis

The protocol is intended primarily for authentic veterinary clinical learning environments in which clinical action, animal behavior, client communication, material objects, and supervision or coaching are visible and consequential. It is especially appropriate for advanced veterinary students in clinical rotations and the clinicians, educators, or supervisors who coach them. In these contexts, the protocol can be used to examine how learners participate in clinical encounters, how educators interpret and coach performance, and how animal patients, clients, tools, space, and team coordination shape clinical learning.

The protocol may also be adapted for interns, residents, practicing veterinarians, veterinary technicians or nurses, and interprofessional clinical teams. However, the intended user group should be specified before data collection because learner autonomy, supervisory responsibility, decision-making authority, communication expectations, and coaching needs differ across educational and professional levels.

For this protocol, a “clinical case” does not necessarily refer to an entire diagnostic workup, hospitalization, surgical procedure, or treatment episode. The primary unit of analysis is a bounded clinical interaction or interactional sequence within a veterinary encounter. Suitable focal cases include moments in which clinical action changes direction, animal behavior alters the flow of care, a client contribution becomes consequential, a learner receives coaching, a material object enables or constrains action, or a team reorganizes its work.

Examples of suitable focal activities include intake and history-taking, physical examination, weighing or positioning, animal handling or restraint, client communication, treatment planning, student case presentation, discharge communication, mobile or community outreach encounters, and moments of clinical uncertainty or redirection. The protocol is less suitable for urgent emergencies, highly sensitive client conversations, procedures where recording could compromise sterility or safety, or encounters in which recording may interfere with patient welfare, client comfort, or clinical workflow. In such situations, recording should be modified, delayed, or stopped.

### Participants and roles

The primary participants for educational applications are advanced veterinary students in clinical rotations and the clinicians, educators, or supervisors responsible for their coaching. Depending on the research purpose and clinical setting, additional participants may include veterinarians, interns, residents, veterinary technicians or nurses, clients, social workers, animal care staff, volunteers, and other members of the veterinary team. Animal patients are not treated as interview participants, but their behavior, movement, posture, vocalization, cooperation, resistance, and interaction with humans and objects are central to the observational record.

The protocol requires at least three methodological roles. The clinical actor participates in or leads the veterinary interaction. The coach/facilitator guides rapid playback, reflection, and assessment. The researcher/observer manages recording, fieldnotes, data organization, and later analysis. In some settings, the same person may serve as both coach and researcher; however, this should be documented because it affects reflexivity and interpretation.

### Ethical and data governance procedures

Because the protocol involves audiovisual recording of clinical work, human participants, clients, students, and potentially identifiable clinical environments, ethics approval should be obtained before implementation. Consent procedures should specify who will be recorded, how recordings will be used, whether recordings will be used for research, education, quality improvement, or all three, and whether participants may withdraw from recording or request deletion of specific footage.

For animal patients, investigators should follow institutional animal care, clinical governance, and welfare requirements. The protocol should not alter the standard of care or delay urgent treatment. Recording and playback should be stopped immediately if they interfere with patient welfare, clinical safety, client comfort, or clinician judgment.

All video files should be stored on encrypted devices or secure institutional servers. File naming should avoid client or patient identifiers. When possible, research transcripts should replace names with role labels such as “clinician,” “student,” “client,” “technician,” “coach,” or “patient.” Because de-identification of video data is difficult, access to raw footage should be restricted to authorized members of the research or educational team.

### Preparation before data collection

Before using the protocol, the team should define the purpose of the assessment. For example, the project may focus on student learning, client communication, animal handling, patient welfare, team coordination, clinical decision-making, coaching practice, or quality improvement. The team should also identify the clinical site, participant groups, consent procedures, camera placement rules, data storage plan, and criteria for selecting candidate sequences.

The coach/facilitator should prepare a short set of reflective prompts. These prompts should be open-ended and should avoid assigning blame or prematurely interpreting participant intention. Examples include:

- “When were you at your best?”
- “What did you notice happening in this moment?”
- “What changed in the interaction after this point?”
- “How did the animal's behavior shape what you did next?”
- “What role did the equipment, space, or object play here?”
- “What were you trying to accomplish clinically?”
- “What might you do similarly or differently next time?”

The team should also try different camera angles, audio quality, playback process, and file transfer procedures before collecting formal data.

*Estimated preparation time:* 1 h for site preparation and team briefing; 10–20 min before each recording session for equipment setup.

*Pause point:* The protocol can pause after site preparation if consent, equipment testing, or clinical scheduling is incomplete.

#### Step 1: record naturally occurring clinical interaction with Camera 1

Camera 1 should be positioned to capture the clinical interaction with minimal disruption to care. The camera should record the human participants, animal patient, relevant clinical objects, and as much of the interactional space as possible. Camera placement will vary by setting. In an examination room, a fixed wide-angle camera may be sufficient. In mobile, farm, or shelter settings, a handheld or tripod-mounted camera may be more appropriate. In high-noise environments, an external microphone may be required.

The recording should begin before the focal interaction starts, when feasible, to capture how the encounter is organized. For example, recording may begin as the client enters the examination room, as the student prepares equipment, or as the patient is positioned for examination. Recording should continue until the focal activity ends or until clinical circumstances make recording inappropriate.

During recording, the researcher should take brief fieldnotes documenting the date, site, participants by role, type of encounter, patient species, relevant objects, and any visible disruptions or contextual factors. The researcher should avoid interrupting clinical care unless there is a safety, consent, or welfare concern.

*Estimated time:* 5–60 min per clinical encounter, depending on the setting and clinical activity.

*Pause point:* Stop recording if a participant withdraws consent, if urgent care is required, if recording interferes with clinical work, or if the animal patient shows distress that requires clinical attention.

#### Step 2: identify candidate sequences for rapid playback

After or during the clinical encounter, the researcher, coach, or clinical educator identifies short candidate sequences for rapid playback. Candidate sequences are moments in which the interaction appears to shift, stall, redirect, intensify, or become clinically or educationally significant. These may include moments when an animal patient resists or cooperates, when a clinician changes strategy, when a client offers important information, when a student receives feedback, when a material object changes what is possible, or when the team reorganizes its work.

Candidate sequences should be short enough to support focused reflection. In most cases, clips of 30 s to 3 min are appropriate. Longer clips may be used when the interactional pattern requires more context.

Selection should remain descriptive at this stage. The researcher should avoid assuming why the sequence mattered before participants have had the opportunity to interpret it. For example, rather than labeling a clip “student mishandles patient,” the researcher might label it “patient movement during weighing” or “change in handling strategy.”

*Estimated time:* 5–10 min per encounter for initial sequence identification.

*Pause point:* If rapid playback is not possible because of clinical workload, participant availability, or patient needs, clips may be marked for later same-day review.

#### Step 3: conduct rapid playback, coaching, and reflexive assessment

The selected Camera 1 footage is replayed to relevant participants as soon as feasible after the clinical interaction. The aim is to support reflection while the encounter is still fresh. The playback may occur in a quiet corner of the clinic, a teaching room, a mobile clinic workspace, or another private location that does not disrupt care.

The coach/facilitator guides the discussion using open-ended prompts. Participants are invited to describe what they noticed, what they were trying to accomplish, how they interpreted the animal patient's behavior, how client communication shaped the encounter, and how tools or space influenced what happened. The coach may also invite participants to identify alternative actions, teaching points, or quality improvement opportunities.

The coaching process should focus on learning and sense-making rather than evaluation alone. The facilitator should avoid framing the recording as evidence of error unless the project is explicitly designed for safety investigation and appropriate safeguards are in place. In educational contexts, the playback should help participants notice tacit clinical judgment, embodied handling skills, communication strategies, and contextual adaptations.

*Estimated time:* 5–20 min per playback session.

*Pause point:* The playback session can be paused or stopped if participants become uncomfortable, if clinical duties require their return, or if the discussion risks breaching client confidentiality in an inappropriate setting.

#### Step 4: record coaching and reflexive sense-making with Camera 2

Camera 2 records the playback, coaching, and reflexive assessment session. This second recording captures the interpretive work that follows the clinical encounter. It documents how participants explain the interaction, how the coach structures reflection, how feedback is delivered, and how clinical practice is translated into teachable or improvable elements.

Camera 2 should capture both the playback interaction and the participants' discussion where possible. The recording should include the coach's prompts, participant responses, moments of agreement or disagreement, and any proposed practice changes. If the original Camera 1 footage is visible in the Camera 2 frame, the researcher should ensure that this does not create additional privacy risks beyond the approved protocol.

Camera 2 is especially important for codifying veterinary coaching. Analysis of Camera 2 data can identify recurring coaching practices such as prompting observation, asking for clinical reasoning, inviting attention to animal behavior, linking action to patient welfare, naming communication strategies, identifying material constraints, and translating reflection into future action. Over time, these data can be used to develop coaching frameworks, faculty development resources, student assessment rubrics, or clinical teaching guides.

*Estimated time:* same duration as playback session, usually 5–20 min.

#### Step 5: pair and organize the two data streams

After each session, Camera 1 and Camera 2 files should be paired using a consistent naming convention. For example:

*Site_Date_CaseNumber_Camera1_ClinicalInteraction*

*Site_Date_CaseNumber_Camera2_CoachingReflection*

The researcher should create a case log that links the two recordings and records the focal activity, participants by role, patient species, length of encounter, selected candidate sequence, timing of playback, and preliminary analytic notes. The case log should also identify any consent restrictions, technical problems, missing audio, or contextual factors that may affect interpretation.

The paired structure is essential. Camera 1 shows what happened in clinical practice. Camera 2 shows how participants interpreted, coached, validated, challenged, or operationalized what happened. Analysis should preserve the relationship between the two recordings.

*Estimated time:* 10–20 min per case for file transfer, naming, and logging.

*Pause point:* Analysis can pause after secure upload and case logging.

#### Step 6: transcribe and prepare selected sequences for analysis

Selected sequences should be transcribed at a level of detail appropriate to the research purpose. For studies focused on broad educational or quality improvement patterns, a verbatim transcript with time stamps and fieldnote annotations may be sufficient. For studies focused on interactional detail, the transcript should include pauses, overlaps, gestures, gaze, animal movement, object use, and changes in spatial arrangement.

Transcription should include both verbal and non-verbal features where relevant. In veterinary practice, important interactional data may include the animal patient pulling away, leaning toward a handler, orienting toward food, vocalizing, freezing, retreating, accepting touch, or changing posture. Material features should also be noted, such as the use of a leash, muzzle, towel, scale, treat, examination table, carrier, syringe, or electronic medical record.

The transcript should preserve time stamps linking the Camera 1 sequence to the Camera 2 coaching discussion. This allows researchers to compare the recorded interaction with participants' subsequent interpretations.

*Estimated time:* 30–120 min per selected sequence, depending on length and level of detail.

#### Step 7: conduct close interactional analysis

Close analysis begins with the Camera 1 clinical interaction. Researchers should examine how the sequence unfolds moment by moment. Analytic attention should be given to what changes, who or what contributes to the change, and how participants respond. The analysis should consider human communication, animal behavior, object use, spatial arrangement, timing, clinical goals, and educational context.

The following analytic questions can guide review:

- What is happening immediately before the focal moment?
- What changes in the interaction?
- How do clinicians, students, clients, technicians, or animal patients respond?
- What material objects or spatial arrangements shape the available actions?
- How is clinical judgment displayed?
- How is patient welfare protected or negotiated?
- How does the interaction become a teaching or coaching opportunity?
- What practice could be repeated, refined, or avoided?

Camera 2 is then analyzed to understand how participants make sense of the same sequence. Researchers should compare what is visible in the Camera 1 recording with what participants identify during playback. This comparison provides interactional validation. It also helps avoid over-attributing intention to animal patients or material objects. Rather than claiming that a non-human actor “intended” an outcome, the analysis should describe how animal behavior or material arrangements influenced the flow of clinical action.

Analytical methods should be selected based on the study aim. Suitable approaches include interaction analysis, reflexive thematic analysis, video-reflexive ethnographic analysis, multimodal analysis, qualitative content analysis, or framework analysis. If the project is designed for quality improvement, findings may also be organized into practice recommendations, process maps, implementation barriers, or coaching tools.

#### Step 8: validate interpretations

Validation occurs through several mechanisms. First, rapid playback allows participants to interpret the clinical sequence soon after it occurs. Second, Camera 2 preserves those interpretations as data. Third, researchers can compare participant interpretations with the audiovisual record. Fourth, preliminary findings may be reviewed with educators, clinicians, or participants to assess whether the analysis is recognizable and useful.

Validation should not be understood as simple agreement among participants. Disagreement may be analytically valuable, especially when clinicians, students, clients, or technicians interpret the same interaction differently. For example, a clinician may view an animal's movement as resistance, while a client may interpret it as fear, pain, excitement, or a known behavioral pattern. These differences should be documented because they reveal how veterinary clinical practice is negotiated among multiple perspectives.

#### Step 9: operationalize coaching practices

A distinctive feature of this protocol is that it treats veterinary coaching as an object of analysis rather than only as an educational intervention. Camera 2 data should therefore be reviewed to identify how coaching is enacted. Researchers may code coaching moves such as asking participants to notice, inviting clinical reasoning, naming an interactional pattern, linking behavior to welfare, connecting action to client communication, demonstrating an alternative, prompting reflection, or translating reflection into future practice.

Over multiple cases, these coaching moves can be organized into a coaching framework. For example, analysis may reveal recurring coaching practices such as:

1. Observation prompts, where the coach asks participants to describe what they see before interpreting it.
2. Clinical reasoning prompts, where the coach asks why a particular action was chosen.
3. Animal-attunement prompts, where the coach directs attention to patient behavior or welfare.
4. Material-awareness prompts, where the coach asks how tools, space, or equipment shaped action.
5. Communication prompts, where the coach links clinical action to client understanding or trust.
6. Future-action prompts, where the coach helps participants identify what they would repeat or change.
7. Leadership prompts, where the coach helps participants see how larger purpose, alignment, and commitments shift process and practice over time.

These categories can be refined inductively from the data. The goal is to make veterinary coaching visible, teachable, and reproducible.

#### Step 10: example application

One example involves a canine patient who resists movement onto a scale during a clinical encounter. Camera 1 records the clinician, client, technician, dog, leash, scale, and dog treat as the interaction unfolds. The dog pulls away from the scale, the client is unable to redirect the dog, and the clinician requests a treat. The clinician then uses the treat to gain the dog's attention, reduce resistance, and guide the dog onto the scale. In the Camera 1 recording, the treat is not merely a background object; it becomes part of the clinical strategy that changes the possible next action.

The Camera 1 footage is then replayed to the clinician and, where appropriate, the student, technician, client, or supervisor. During playback, the coach asks: “What did you notice happening when the treat was introduced?” “What were you trying to accomplish?” “How did the dog's behavior change?” “How did the treat alter what was clinically possible?” Camera 2 records this coaching and reflection. The resulting paired data allow the research team to analyze both the original interaction and the coaching process through which the clinician explains and refines the practice.

This example may generate several outputs: a validated description of treat-assisted redirection, a teaching point about low-stress handling, a coaching prompt for student learning, and a quality improvement opportunity related to preparing exam rooms with appropriate animal-handling resources.

### Observable indicators and analytic outputs

The protocol is designed to generate observable indicators of clinical learning, coaching, professional development, and practice improvement. These indicators should be selected according to the purpose of the study rather than assumed to occur in every case. Learning indicators may include moments when a learner identifies what changed in an encounter, explains clinical reasoning, recognizes the influence of animal behavior, connects communication to client response, or proposes a future adjustment. Coaching indicators may include educator prompts that direct attention to observation, clinical reasoning, animal welfare, client communication, material constraints, team coordination, or future action. Professional development indicators may include participant reflection on role responsibility, uncertainty, judgment, communication, teamwork, or emerging professional identity. Clinical practice indicators may include identification of a repeatable technique, recognition of a workflow problem, refinement of an animal-handling strategy, or documentation of a quality improvement opportunity.

Scientific knowledge is generated by transforming paired Camera 1 and Camera 2 recordings into participant sense- and meaning-making of interactional cases. Camera 1 data document what occurred in the clinical encounter. Camera 2 data document how participants interpreted, validated, questioned, or reframed that encounter during coaching and reflection. Researchers can then code recurring interactional patterns, coaching moves, clinical adaptations, animal responses, material constraints, and participant interpretations across cases. Cross-case comparison allows researchers to identify patterns that are local enough to remain clinically meaningful but sufficiently organized to inform veterinary education, coaching frameworks, clinical communication research, One Welfare-oriented practice, or quality improvement. In this way, the protocol moves from description to analysis by linking observed interaction, participant interpretation, coding, validation, and synthesis into transferable methodological and educational knowledge.

### Data outputs

The protocol produces the following outputs:

- Paired audiovisual recordings of clinical interaction and coaching reflection.
- Time-stamped case logs linking clinical sequences to coaching discussions.
- Fieldnotes documenting setting, participants, patient species, and contextual factors.
- Transcripts of selected clinical and coaching sequences.
- Participant-validated interpretations of focal interactions.
- Analytic summaries of human-animal-material interaction patterns.
- Codified coaching moves and educational prompts.
- Practice recommendations for teaching, clinical improvement, or quality improvement.

### Replicability and quality control

To support replicability, researchers should document camera placement, participant roles, consent procedures, sequence selection criteria, playback timing, coaching prompts, transcription conventions, analytic method, and validation procedures. A standardized case log and coaching prompt sheet should be used across cases. When multiple researchers are involved, a subset of sequences should be reviewed jointly to calibrate sequence identification and analysis.

Because this is a qualitative and interactional protocol, precision and accuracy are not measured through statistical limits of detection. Instead, rigor is supported through transparent documentation, preservation of audiovisual data, participant validation, paired analysis of clinical and coaching recordings, reflexive fieldnotes, and clear reporting of analytic decisions. Results are replicable to the extent that other teams can follow the same recording, playback, coaching, pairing, transcription, and analysis procedures in comparable veterinary clinical settings.

### Safety, welfare, and interruption rules

Clinical care takes priority over data collection. Recording, playback, or coaching should not delay urgent treatment, increase animal distress, compromise safety, or interfere with clinician judgment. The protocol should include explicit interruption rules. Any participant may request that recording stop. The clinician responsible for the patient may stop recording or playback at any time. The coach or researcher should also stop the protocol if the recording process becomes disruptive.

### Timing summary

A complete cycle can be conducted within a single clinical session. Equipment setup typically requires 10–20 min. Camera 1 recording lasts 5–60 min, depending on the encounter. Candidate sequence selection requires 5–10 min. Playback and coaching require 5–20 min. File pairing and logging require 10–20 min. Transcription and close analysis occur later and vary by project scope.

In practice, a brief case may be recorded, reviewed, and logged in under 1 h. More complex cases, especially those used for research publication or faculty development, require additional time for transcription, analysis, validation, and team review.

## (Anticipated) Results

Because this article presents a protocol rather than the findings of a completed empirical study, the results described here are the anticipated outputs of applying the two-camera participatory Rapid Ethnographic Assessment protocol in veterinary clinical settings. The expected outcome is not a singleI quantitative measurement but a structured set of audiovisual, reflective, educational, and analytic products that can be used to study and improve clinical practice. Similar video-reflexive approaches in healthcare have shown value for making everyday clinical work visible, supporting practitioner reflection, and identifying opportunities for local practice improvement (9, 10, 22, 28). In veterinary clinical practice, the protocol is expected to produce comparable forms of insight while also attending to the distinctive role of animal patients, client relationships, and material objects in shaping the encounter.

### Expected protocol outputs

The protocol is expected to generate four primary outputs.

First, it should produce audiovisual records of naturally occurring veterinary clinical interactions. Camera 1 recordings should capture how clinicians, students, technicians, clients, animal patients, and material objects interact during clinical care. These recordings provide an observable account of real-time practice rather than relying only on retrospective interviews or self-report.

Second, the protocol should produce participant-reflected accounts of those interactions. Through rapid playback, participants are expected to identify features of practice that may otherwise remain tacit, including clinical judgment, handling strategies, client communication, affective responses, environmental constraints, and animal behavioral cues. These reflexive accounts serve as interactional validation by allowing participants to explain what they noticed, intended, adjusted, or learned during the encounter.

Third, the protocol should produce audiovisual records of veterinary coaching. Camera 2 recordings should capture the coaching, feedback, questioning, and interpretive work that occurs during playback. These recordings make it possible to analyze how veterinary educators and supervisors guide reflection, name clinical patterns, prompt attention to animal behavior, and translate clinical experience into future action. Over time, this output can support the codification and operationalization of veterinary coaching practices.

Fourth, the protocol should produce practice-development and quality-improvement insights. The paired Camera 1 and Camera 2 data should help teams identify repeatable practices, recurring interactional challenges, material constraints, communication patterns, and educational opportunities. These insights may be translated into teaching resources, coaching guides, clinical checklists, workflow adjustments, or quality improvement aims.

### Anticipated data products

Application of the protocol should result in the following data products:

- Paired Camera 1 and Camera 2 video files.
- Case logs linking clinical interactions to coaching sessions.
- Time-stamped candidate sequence records.
- Fieldnotes describing setting, participant roles, patient species, relevant objects, and contextual factors.
- Transcripts of selected clinical interaction sequences.
- Transcripts of selected coaching and reflexive assessment sequences.
- Analytic memos comparing what occurred in practice with how participants interpreted the event.
- A catalog of coaching prompts and coaching moves.
- A list of human-animal-material interaction patterns relevant to clinical teaching or improvement.
- Optional quality improvement outputs, such as process maps, teaching checklists, or implementation recommendations.

These outputs provide the evidentiary basis for qualitative analysis, participant-validated interpretation, coaching-framework development, and practice improvement. They allow researchers to move beyond description by comparing what occurred in practice with how participants interpreted the event, how coaching shaped reflection, and how similar interactional patterns recur across clinical cases.

### Observable indicators and analytic interpretation

In addition to producing audiovisual and textual data, the protocol is expected to make specific indicators of learning, coaching, professional development, and practice improvement observable. Learning indicators may include moments when a learner identifies what changed in an encounter, explains clinical reasoning, recognizes the influence of animal behavior, connects communication to client response, or proposes a future adjustment. Coaching indicators may include educator prompts that direct attention to observation, clinical reasoning, animal welfare, client communication, material constraints, team coordination, or future action. Professional development indicators may include participant reflection on role responsibility, uncertainty, judgment, communication, teamwork, or emerging professional identity. Clinical practice indicators may include identification of a repeatable technique, recognition of a workflow problem, refinement of an animal-handling strategy, or documentation of a quality improvement opportunity.

The paired data structure is expected to support scientific knowledge generation by linking observed interaction, participant interpretation, coding, validation, and cross-case synthesis. Camera 1 data document what occurred in the clinical encounter. Camera 2 data document how participants interpreted, validated, questioned, or reframed that encounter during coaching and reflection. Researchers can then compare the clinical sequence with the coaching sequence, code recurring interactional patterns and coaching moves, and examine similarities and differences across cases. This process can generate empirically grounded categories, typologies of human-animal-material interaction, coaching frameworks, practice recommendations, and hypotheses for future assessment, educational, or quality improvement studies.

### Illustrative expected outcome

An expected outcome can be illustrated using the manuscript's example of a dog treat used during clinical care. In this example, Camera 1 records a clinical sequence in which a canine patient resists movement onto a scale. The clinician requests a treat, uses it to redirect the dog's attention, and successfully guides the dog onto the scale. The resulting footage captures not only the clinician's action but also the interaction among the dog, client, technician, leash, scale, treat, and clinical space.

During rapid playback, Camera 1 footage is reviewed with relevant participants. The coach may ask what the clinician noticed, why the treat was introduced, how the dog's behavior changed, and how the material object altered what was clinically possible. Camera 2 records this discussion. The anticipated result is a paired data sequence showing both the clinical interaction and the subsequent coaching process through which participants interpret and operationalize the practice.

From this single sequence, the protocol may produce several usable results: a teachable example of low-stress redirection, a coaching prompt for student learning, an analytic memo about material objects in clinical practice, and a quality improvement recommendation to make appropriate handling aids readily available in examination spaces.

### Suggested figures

*Figure 1. Two-camera participatory REA workflow*.

This figure should show the movement from Camera 1 recording of clinical interaction, to rapid playback, to Camera 2 recording of coaching/reflection, to paired analysis and practice improvement.

*Figure 2. Paired data structure*.

This figure should illustrate how one clinical sequence from Camera 1 is linked to the corresponding Camera 2 coaching sequence, transcript, fieldnote, and analytic memo.

*Figure 3. Example human-animal-material interaction map*.

This figure could use the dog-treat example to show the clinician, dog, client, technician, treat, leash, and scale as interacting components that redirect the clinical encounter.

### Advantages of the protocol

The protocol has several anticipated advantages.

First, it captures veterinary clinical practice as it unfolds. This is important because many features of practice are embodied, relational, spatial, and time-sensitive. Video allows researchers and educators to revisit details that may be missed during live observation.

Second, the protocol links observation with participant interpretation. Rapid playback allows participants to explain what mattered in the moment, reducing the risk that researchers impose interpretations without reference to practitioner or client understanding.

Third, the two-camera structure makes coaching visible. Rather than treating coaching as a background educational activity, Camera 2 records how coaching is enacted. This creates a dataset that can be used to study, improve, and teach veterinary coaching.

Fourth, the protocol can support both scholarship and practice improvement. It can generate data for qualitative research while also producing local learning resources, faculty development materials, and quality improvement insights.

Fifth, the protocol is adaptable when investigators specify the intended user group, clinical context, and unit of analysis before data collection. It is especially suitable for bounded clinical interactions in which animal behavior, client communication, learner participation, material objects, and coaching are visible and consequential. Broader applications across teaching hospitals, private practice, shelter medicine, community outreach, mobile clinics, farm animal practice, equine practice, or simulation should be justified according to the study purpose and local workflow.

### Limitations

The protocol also has limitations. Video recording may influence participant behavior, especially during early use. Clinicians, students, or clients may act differently when they know they are being recorded. This reactivity may decrease as participants become familiar with the process, but it should be acknowledged in the analysis.

The protocol is also time-intensive. Although rapid playback can occur within minutes, transcription, coding, and paired analysis require substantial researcher time. Projects using this method should carefully define the number of cases and sequences needed to answer the research or educational question.

Another limitation is that video data are difficult to anonymize. Even when names are removed from transcripts, raw video may contain identifiable faces, voices, clinical spaces, clients, staff, and animal patients. This creates privacy and data governance challenges.

The protocol does not allow researchers to determine animal intention. It can document animal behavior and how that behavior shapes clinical action, but it should not over-interpret the animal patient's internal state. Participant interpretation, client knowledge, clinical expertise, and careful description should be used to avoid anthropomorphic or unsupported claims.

Finally, the protocol is context-sensitive. Findings from one clinic, species, specialty, or educational environment may not transfer directly to another. Replicability depends on transparent reporting of the setting, participants, camera placement, coaching process, analytic method, and validation procedures.

### Troubleshooting guidance

If playback cannot occur immediately after the clinical interaction, the footage should be marked for same-day review. The interpretive value of the protocol is strongest when participants still remember the encounter; however, delayed playback may still be useful if the footage is clear and the case log contains sufficient contextual detail.

If the clinical environment is too crowded or noisy for Camera 1, researchers should prioritize audio capture and the most relevant interactional space. For example, in a busy treatment area, the camera may focus on the clinician, animal patient, and immediate handling context rather than the entire room.

If participants appear uncomfortable during playback, the coach should pause the video and restate the purpose of the process as learning, reflection, and practice improvement. Participants should be reminded that they may decline to comment on a sequence or request that a clip not be used for research or teaching, consistent with the approved consent process.

If the coaching discussion moves too quickly into evaluation, the coach should return to descriptive prompts: “What do you notice?” “What changed here?” “What options were available at this moment?” These prompts help preserve the protocol's emphasis on interactional sense-making rather than judgment.

If analysis produces disagreement among participants, the disagreement should be retained as data. Differences between clinician, student, client, technician, and coach interpretations may reveal important features of veterinary clinical practice, including hidden assumptions, communication gaps, or species-specific knowledge.

### Criteria for successful implementation

The protocol can be considered successfully implemented when it produces: paired clinical and coaching recordings; clear linkage between Camera 1 and Camera 2 data; participant reflection on selected sequences; usable descriptions of human-animal-material interaction; identifiable coaching practices; and at least one educational, analytic, or quality improvement output.

A strong implementation should also demonstrate that the protocol did not interfere with clinical care, compromise animal welfare, or undermine participant trust. Successful use of the method depends not only on data quality but also on the creation of a psychologically and ethically safe environment for reflection.

### Anticipated contribution of results

The anticipated results should help researchers, practitioners, and educators move from general claims about the complexity of veterinary practice to concrete, observable, and teachable examples. The protocol should make visible how clinical action is shaped by human judgment, animal behavior, client participation, material objects, and environmental constraints. It should also provide a structured way to study veterinary coaching as a practice in its own right.

In this way, the expected results are both scholarly and practical: the method generates analyzable qualitative data while also supporting clinical teaching, reflexive learning, and context-sensitive practice improvement.

## Discussion

This Methods article presents a participatory Rapid Ethnographic Assessment protocol for studying, teaching, and improving veterinary clinical practice. The protocol responds to a practical methodological problem: veterinary clinical encounters are complex, fast-moving, interspecies, materially situated, and educationally rich, yet many available methods rely on retrospective accounts or observations that do not fully capture how practice unfolds moment by moment. By pairing video-recorded clinical interaction with video-recorded coaching and reflexive assessment, the protocol creates a structured way to examine both what happens during care and how participants interpret, teach, and operationalize that care immediately afterward.

The central contribution of the protocol is its two-camera design. Camera 1 captures naturally occurring clinical interaction and provides the footage used for rapid playback. Camera 2 captures the coaching, reflection, and interpretive discussion that follows. This pairing allows researchers and educators to connect clinical action with situated participant meaning-making. It also extends the current manuscript's original methodological focus on human, animal, and material contributions to practice while shifting the emphasis toward a practical protocol for veterinary educators, practitioners, and researchers.

### Contribution to veterinary clinical education

Veterinary education is increasingly understood as workplace-based, situated, social, and practice-dependent rather than simply the transfer of technical knowledge from expert to learner. Clinical competence develops as students and new practitioners learn to notice relevant cues, coordinate with others, respond to uncertainty, communicate with clients, handle animals safely, and adapt to changing clinical circumstances. Qualitative research in veterinary education has emphasized the importance of studying clinical learning in real practice settings, where professional judgment, identity, and communication are formed through participation in everyday work (3, 4, 7, 29).

The protocol contributes to this work by making clinical learning visible. Rather than relying only on *post hoc* student reflection or supervisor assessment, it allows educators and learners to review specific moments of practice together. This supports more precise feedback because the discussion is anchored in a shared audiovisual record. The approach may be particularly useful for teaching skills that are difficult to capture through checklists alone, such as clinical presence, timing, embodied animal handling, client attunement, team coordination, and adaptive judgment.

The second camera is especially important for veterinary education because it captures the coaching process itself. Coaching is often treated as an informal or tacit part of clinical teaching. This protocol turns coaching into analyzable data. Over time, Camera 2 recordings can help educators identify recurring coaching moves, refine prompts, compare supervisory approaches, and develop faculty development resources. In this way, the protocol supports not only student learning but also the improvement of veterinary teaching practice.

### Contribution to veterinary humanities and social sciences

The protocol is also well aligned with veterinary humanities and veterinary social science because it treats clinical practice as more than biomedical decision-making. Veterinary encounters involve relationships among humans, animals, technologies, objects, spaces, institutional expectations, economic constraints, and ethical commitments. Veterinary humanities and social science approaches are increasingly concerned with how veterinary work is experienced, organized, narrated, embodied, and socially situated.

This protocol offers a practical method for studying those dynamics empirically. It allows researchers to examine how clinical interactions are shaped by animal behavior, client knowledge, professional authority, material tools, and space. For example, a leash, scale, table, muzzle, carrier, consent form, treat, or electronic medical record may become consequential in the unfolding of care. The protocol does not require researchers to make speculative claims about the intentions of animals or objects. Instead, it supports careful analysis of observable interaction: what changed, who or what contributed to the change, how participants interpreted it, and how the team responded.

This is especially valuable for multispecies and more-than-human research. The method allows animal patients to be taken seriously as participants in clinical encounters without anthropomorphizing them. Their movements, resistance, cooperation, posture, vocalization, and orientation can be analyzed as part of the interactional field, while participant reflection provides a check against over-interpretation.

### Contribution to One Welfare and context-sensitive practice

The protocol may also support One Welfare-oriented research and practice. One Welfare emphasizes the interconnectedness of animal welfare, human wellbeing, social conditions, and environmental context (15–17). Veterinary clinical encounters often reveal these interconnections in concrete ways. A patient's behavior may affect a clinician's handling strategy; a client's social situation may shape treatment feasibility; a clinic's spatial arrangement may influence patient stress; and available tools may determine whether a low-stress or welfare-sensitive option is possible.

By capturing these interactions in real time, the protocol can help teams identify how welfare-relevant practices are actually enacted. It can be used to study how clinicians reduce fear, stress, or conflict; how clients contribute knowledge about animal behavior; how material arrangements support or hinder humane handling; and how clinical teams adapt care to social or environmental constraints. This makes the method useful not only for education and research but also for local improvement efforts aimed at strengthening patient welfare and client-centered care.

### Relationship to video-reflexive and rapid ethnographic methods

The protocol builds on established strengths of rapid ethnographic assessment and video-reflexive ethnography. Rapid ethnographic approaches are useful when researchers need context-sensitive insight within practical time frames. Video-reflexive ethnography has shown that video can help practitioners see everyday work differently, reflect on tacit practices, and identify opportunities for improvement in complex healthcare settings (9, 10, 22, 28).

The protocol adapts these strengths for veterinary clinical practice. Veterinary settings require attention not only to team communication and workflow but also to interspecies interaction. The animal patient is not simply the object of care; the patient's behavior can redirect the encounter, alter clinical options, and become central to learning. The two-camera design therefore extends video-reflexive practice by pairing the clinical sequence with the coaching and sense-making sequence. This structure allows researchers to analyze both the encounter and the educational process through which the encounter becomes meaningful.

### Practical applications

The protocol has several practical applications. In veterinary teaching hospitals, it can be used to support clinical teaching, student assessment, faculty development, and professional identity formation. In general practice or specialty settings, it can support team reflection on client communication, handling practices, workflow, and patient experience. In shelter medicine, mobile clinics, community outreach, or field-based care, it can help researchers and practitioners examine how constrained environments, client vulnerability, limited resources, and human-animal bonds shape care. In simulation, the protocol can be used to compare simulated performance with real clinical encounters or to train students in reflective practice before they enter high-stakes clinical settings.

The protocol can also support quality improvement. Recurrent patterns identified across cases may reveal improvement opportunities such as room setup, availability of handling aids, communication routines, team handoff processes, student supervision practices, or client education materials. Because the method produces participant-validated data, resulting recommendations may be more locally credible and easier to implement than externally imposed interventions.

### Ethical and methodological considerations

The major ethical challenge of the protocol is the use of audiovisual data. Video captures identifiable people, voices, clinical spaces, and animal patients. Consent must therefore be treated as an ongoing process rather than a one-time form. Participants should be able to decline recording, stop recording, request that a clip not be used, or withdraw within the limits of the approved protocol. The use of video for research, education, and quality improvement should be clearly distinguished during consent.

Animal welfare must remain central. The protocol should never delay urgent care, increase patient stress, or interfere with clinical judgment. Recording equipment should be positioned to minimize disruption, and the clinical team should retain authority to stop data collection at any point.

Methodologically, the researcher's role also requires reflexivity. The presence of cameras, the selection of clips, and the framing of coaching questions all influence the data produced. This is not a weakness unique to this protocol; it is a feature of participatory qualitative research. However, it should be made visible through fieldnotes, transparent reporting, and careful documentation of analytic decisions.

## Limitations

The protocol has several limitations. First, it requires time, equipment, consent procedures, and data-management capacity. Clinics with limited staffing or high caseloads may find it difficult to conduct rapid playback during busy periods. Second, video data can be difficult to store, secure, de-identify, and share. Third, participants may initially behave differently when recorded. Familiarization sessions and repeated use may reduce this effect but cannot eliminate it entirely.

Fourth, the protocol produces context-specific qualitative data. Its purpose is not to generate universally generalizable findings in the statistical sense. Rather, it produces transferable insights when researchers provide sufficient detail about setting, participants, clinical activity, species, equipment, and analytic procedures. Fifth, the protocol cannot establish animal intention. It can document animal behavior and analyze how that behavior shapes clinical action, but claims about animal experience should be made cautiously and, where possible, supported by clinical expertise, client knowledge, and welfare science.

Finally, because the protocol combines research, education, and improvement, role clarity is essential. Participants should know whether a recording is being used for research analysis, formative feedback, assessment, quality improvement, or a combination of these purposes. Blurring these purposes may undermine trust.

## Future directions

Future studies should test the protocol across different veterinary contexts, including small animal practice, equine practice, food animal medicine, emergency and critical care, shelter medicine, mobile outreach, and teaching hospitals. Comparative studies could examine how coaching practices differ by setting, species, learner level, or clinical specialty. Researchers could also use the protocol to develop validated coaching frameworks for veterinary clinical education.

Future work should also explore how the protocol can be integrated with existing assessment and quality improvement systems. For example, Camera 2 data could be used to develop faculty development modules, reflective practice curricula, or structured coaching rubrics. Camera 1 data could support research on handling, communication, decision-making, or team coordination. Paired analysis could help identify how specific coaching practices influence learner awareness, confidence, and clinical adaptation.

There is also potential to connect this protocol with implementation science. Once recurring practices are identified—such as treat-assisted redirection, client-supported handling, team communication routines, or low-stress examination-room setup—future studies can examine how those practices are implemented, sustained, and adapted across settings.

## Data Availability

The original contributions presented in the study are included in the article/supplementary material, further inquiries can be directed to the corresponding author.
